# 360-Degree Feedback Model to Enhance Interprofessional Learning

**DOI:** 10.15694/mep.2018.0000154.1

**Published:** 2018-07-25

**Authors:** Linda Awdishu, Amy Zheng, Anne Gerd Granas, Janine Galasso, Karen Macauley, Cheryl Butera, Sophie Hutchins, Peggy Wallace, Karen Garman, Jennifer Namba

**Affiliations:** 1 University of California; 2 Veteran Affairs Office of Inspector General; 3 School of Pharmacy; 4 University of California; 5 University of San Diego; 6 University of California; 7 Kaiser Permanente Southern California

**Keywords:** Interprofessional Education, Feedback, Healthcare Students, Standardized Patients, Simulation

## Abstract

This article was migrated. The article was marked as recommended.

**Background:** Providing meaningful feedback in interprofessional education (IPE) requires knowledge of discipline specific responsibilities and a method for measuring team dynamics while capturing individual performance.

**Methods:** We implemented a 360-degree performance feedback model for a large-scale IPE simulation with standardized patients (SP) who transitioned from primary care to the emergency department. 293 medical, nursing, and pharmacy students were divided into 72 teams. We conducted a retrospective study evaluating feedback from 108 facilitators on individual and team based competencies, 12 SP patient satisfaction surveys and 293 student self-appraisals. We analyzed data using descriptive statistics and ANOVA for multiple group comparisons.

**Results:** More than 94% of SP indicated they would return to the same student team to seek care. However, SP reported that the students did not summarize or clarify information, adapt to their level of understanding or encourage questions. Facilitators noted all disciplines were involved in formulating and implementing treatment plans. Student teams performed highest in mutual support and situational monitoring, and lowest in leadership and team structure. Students across all disciplines rated their teams as requiring light to no supervision.

**Conclusion:** Applying the 360-degree performance model is feasible in IPE and provides multidimensional, qualitative feedback to enhance student learning.

## Introduction

“Interprofessional education (IPE) occurs when two or more professions learn with, from and about each other to improve collaboration and the quality of care.”( CAIPE, 2002) Interprofessional education is increasingly implemented in many health sciences curricula to promote collaboration between disciplines, however, many institutions struggle with developing a process to provide meaningful feedback to student teams. An accurate, valuable and consistent external evaluation and self-appraisal is critical to student learning and professional development. ( Sargeant *et al.*, 2010) This appraisal is central to deliberate practice, in which learning exercises provide immediate feedback, time for problem-solving and evaluation and opportunities for repeated performance. ( Ericsson, 2008) The combination of repeated performance and evaluation is essential to the acquisition of expertise. ( Ericsson, 2004) It is also important for the evaluators to be representative of the multiple professions participating in the activity. Currently, most health sciences programs offer limited interprofessional experiences that are not repeated throughout the curriculum. The feedback provided to students is usually delivered in the form of facilitator-led debriefing sessions immediately following the learning activity. While critical assessment is required for experiential learning, excessive correction or guidance may actually impede the learning process. ( Schmidt and Wulf, 1997) Therefore, it is often a challenge for IPE coordinators to determine the appropriate amount of external feedback necessary for a particular exercise and to strike a balance between too much and too little evaluation.

Another technique used to provide formalized feedback, generally employed by human resource departments, is 360-degree performance feedback. ( Bracken DW, 2011) The performance evaluation is derived from the spectrum of people within an employee’s direct work circle, including supervisors, peers and subordinates. In some cases, it may also include a self-evaluation by the employee, as well as feedback from external sources such as patients. In general, the intent of 360-degree feedback is to help the employee create a path for career development. However, the effect of this model has been mixed due to the variability of assessment models. ( Bracken DW, 2011) In healthcare, the 360-degree model has been applied in both administration and clinical practice for practitioners at varying levels. ( Garman, Tyler and Darnall, 2004; Joshi, Ling and Jaeger, 2004; Overeem *et al.*, 2012) This same concept can be applied to IPE performance appraisal with the combination of feedback from peers, facilitators, and standardized patients, along with a self-assessment. These comprehensive assessments allow students to self-reflect and also compare their own assessment to those of the external evaluators (e.g., facilitators). The 360-degree feedback model has been found to be effective in enhancing IP team performance. (Sikes *et al.*, 2015) By using this approach in IPE evaluations, students may be able to modify their behaviors by incorporating the multifaceted feedback and therefore be better prepared for professional practice. ( Garman, Tyler and Darnall, 2004) The objective of this study is to implement 360-degree performance feedback in IPE and to describe the feedback from facilitators, standardized patients and students.

## Methods

We conducted a retrospective study on the implementation and outcomes of a large-scale IPE simulation involving medical, pharmacy and nursing students. An organizing committee including representatives from each health sciences school (i.e., 2 nurses, 2 pharmacists, 1 physician and 2 medical educators) developed the large-scale IPE.

### Participants

The IPE included second-year medical students from the University of California, San Diego School of Medicine, third-year pharmacy students from the University of California, San Diego Skaggs School of Pharmacy and Pharmaceutical Sciences, and first or second-year nursing students from the University of San Diego, Hahn School of Nursing. Facilitators included faculty members from these respective schools as well as clinicians working in academic and community hospitals in the region. All students and faculty were consented prior to the event. The Institutional Review Boards at the University of California, San Diego and University of San Diego approved this study.

### Setting

The details of the patient case have been previously published. (Zheng A, 2015) In short, the case involves a standardized patient presenting to a primary care clinic with chest pain. The patient has an ST segment elevation myocardial infarction requiring triage by a student team and transfer to the emergency department for a higher level care provided by a second student team. (Zheng A, 2015) There are two simulation settings between which the standardized patient transitions in care: (1) primary care clinic and (2) emergency department. The student team in the primary care clinic simulation includes 1-2 medical students and 1-2 nursing students. The student team in the emergency department (ED) simulation includes 1-2 medical students, 1-2 nursing students and 1-2 pharmacy students. An IP facilitator team (i.e. 1 facilitator from nursing, pharmacy and medicine) observes both student teams and provides feedback to both teams during a debriefing session immediately after the simulation. The primary care medical and nursing students conduct a problem-focused history and physical examination. They work together to diagnose the problem, transfer the patient to the simulated ED while providing hand-off to the second team of students. After the hand-off, the primary care students observe the ED encounter remotely from the debriefing room. The ED student group assesses the patient, develops and implements a treatment plan. At the end of the encounter, the primary care and ED student teams meet with facilitator teams for a debriefing session.

**Figure 1.**
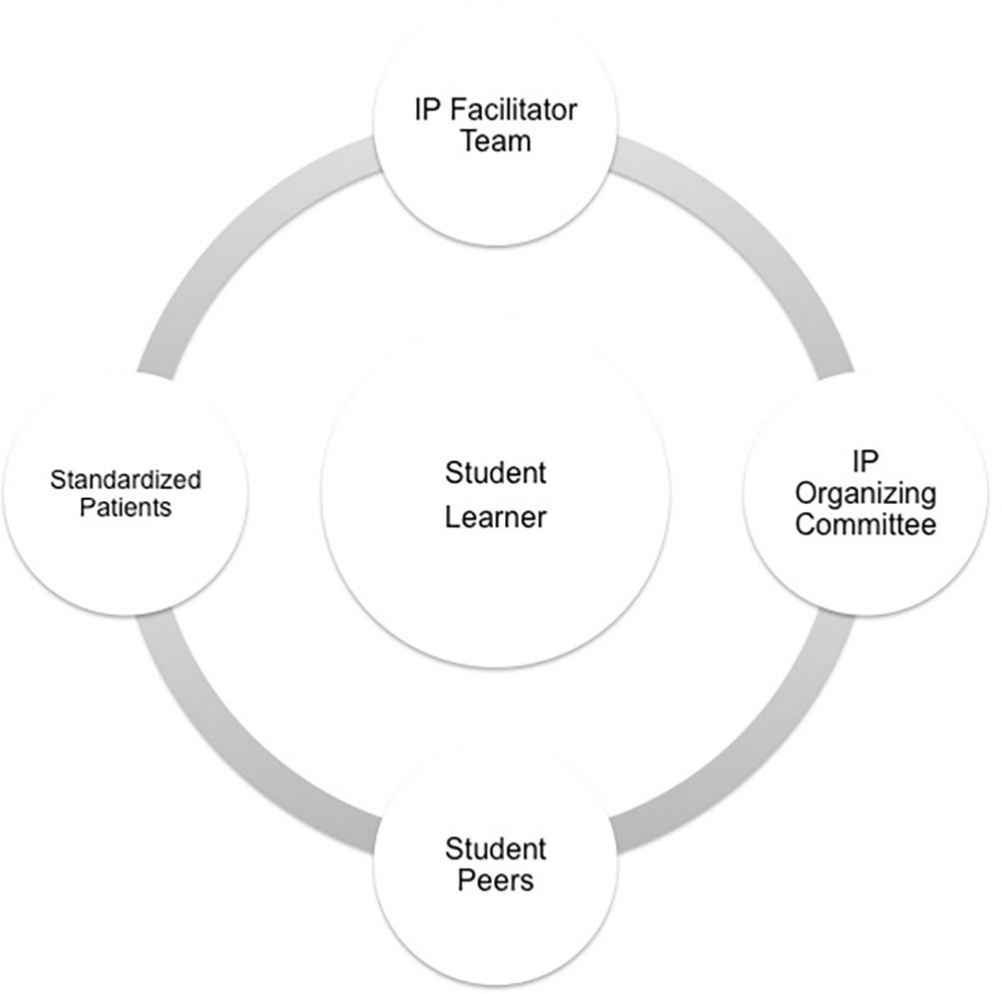
360-Degree Feedback Model

The 360-degree performance feedback model incorporated (1) facilitator assessment of student performance, (2) standardized patient satisfaction surveys, (3) verbal student peer feedback and (4) student self-appraisal ( Figure 1). Verbal student peer feedback occurred during the debriefing sessions but was not captured for this analysis.

### Simulated Patient Training and Checklist

This event was conducted at a simulation center with an established SP program in which SPs undergo extensive training on the case scenario and student evaluation. Use of SPs is superior to high fidelity simulators when assessing student performance in communication domains. (Wallace, 2007) Prior to the event, 12 SPs received training on case portrayal (e.g. emotional state, symptoms of heart attack, etc.) and communication checklist rubric. In order to provide feedback on patient satisfaction, the SPs filled out communication checklists about their experiences with the primary care group and ED group (Appendix A). The SP checklist was adapted by the California Consortium for the Assessment of Clinical Competence from the validated SEGUE framework. (Wallace, 2007; May, 2008)

### Facilitator Training and Checklist

One week prior to the IPE, the facilitators were given a guide which included a checklist encompassing both individual and team-based competencies (Appendix B). The treatment plan was designed using the most recent American College of Cardiology Foundation and American Heart Association guidelines. (O’Gara *et al.*, 2013) The teamwork portion of the checklist originated from the validated Performance Assessment of Communication of Teamwork (PACT) Novice Tool from the University of Washington. (Chiu, Brock and Abu-Rish) The PACT Novice Tool was developed using the TeamSTEPPS ^®^ framework to assess performance on teamwork and communication during simulated events. (Chiu, Brock and Abu-Rish) The facilitator evaluates the student’s performance as a team using the 5 components of TeamSTEPPS ^®^: team structure, leadership, situational monitoring, mutual support and communication. During the facilitator orientation, the organizing committee trained the facilitators on the use of the checklist and the standardized method of post-activity debriefing (discussed further below). The facilitators individually complete the checklist while observing the student teams. It was noted on the first day of the IPE that the PACT tool was not separated for the clinic and ED teams. For the second day of the event, each facilitator rated each team, clinic and ED, separately using the PACT tool.

### Debriefing session

Facilitator teams co-debrief students immediately following the encounter using a structured approach of 3 phases: (1) Student reactions, (2) Analysis of Performance, (3) Summary. In Phase 1, students introduce themselves and discuss one thing they took away from the experience. In Phase 2, IP facilitators review their checklists and facilitate discussion on the team’s knowledge, skills, and attitudes during the patient care scenario. The discussion begins with facilitators asking open-ended questions:

1. What was GOOD about the encounter?

2. What OPPORTUNITIES missed do you need to consider for the next encounter?

3. What did you learn that you did not EXPECT?

In Phase 3 the facilitators summarize key content on the checklists and end the debriefing session discussing the SP feedback with the teams.

### Student Self-Appraisal

After the IPE event, students completed an entrustable professional activity (EPA) evaluation reflecting on their team performance in the competencies of subjective findings, objective findings, team assessment and plan of care (Appendix C). For each competency, descriptors were provided to guide rating (Appendix C). The EPA is typically used by faculty to evaluate the level of supervision required by trainees based on the achievement of competencies. All learners must have a sufficient level of proficiency at the completion of their training. EPA tools determine when students are competent to assume specific clinical responsibilities without supervision. The EPA tool is a global rating scale ranging from zero - critical deficiencies, one - heavy supervision, two - light supervision, three - no supervision needed, and four - aspirational performance.

### IPE Event Evaluation

All students completed an anonymous survey evaluating the IPE event. Students were asked questions about whether the IPE event allowed them to reflect on clinical abilities, recognize clinical strengths and weaknesses, reflect and discuss performance, learn through feedback and the overall value of the event.

### Statistical Analysis

Data were collected from checklists and surveys completed by event participants. Data were analyzed using descriptive statistics and EPA scores between the groups were compared using the Kruskal-Wallis one-way ANOVA test. Post-hoc analysis was performed using pairwise comparison between groups. The statistical analysis was performed using R version 3.4.2.

## Results/Analysis

In total, 12 standardized patients interacted with 36 primary care groups and 36 ED groups to evaluate a total of 293 students. All of the 12 standardized patients, the 108 facilitators and the 293 students completed their respective 360-Degree Performance Feedback Forms (Appendices A, B, C). All scenarios were enacted within the set time limit of 30 minutes per team. No students withdrew from their team.

### Standardized patient feedback

From the 72 standardized patient satisfaction surveys, 97.2% and 94.4% of SPs strongly agreed or agreed that they would return to this primary care clinic or ED, respectively. Overall, the SPs reported that the student teams made a personal connection with the patient. However, in the primary care setting some SPs reported that the student teams did not summarize or clarify information (38.9%), adapt information to their level of understanding using appropriate language (25.0%) or encourage the SP to ask questions (27.8%) ( Table 1). In the ED the SPs felt that the student teams did not encourage them to ask questions (11.1%) ( Table1).

**Table 1.** Standardized Patient Satisfaction Scores for Team Performance

Question	Location ^*^	Agree N(%)	Somewhat N(%)	Disagree N(%)
The healthcare providers made a personal connection during the visit.	Clinic	28 (77.8)	7 (19.4)	1 (2.8)
ED	27 (75.0)	9 (25.0)	0 (0)	
The healthcare providers gave me an opportunity/time to talk.	Clinic	34 (94.4)	1 (2.8)	1 (2.8)
ED	32 (88.9)	4 (11.1)	0 (0)	
The healthcare providers actively listened. Gave me undivided attention.	Clinic	31 (86.1)	5 (13.9)	0 (0)
ED	25 (69.4)	9 (25)	2 (5.6)	
The healthcare providers summarized and/or clarified information.	Clinic	21 (58.3)	1 (2.8)	14 (38.9)
ED	28 (77.8)	8 (22.2)	0 (0)	
The healthcare providers treated me with respect.	Clinic	36 (100)	0 (0)	0 (0)
ED	34 (94.4)	2 (5.6)	0 (0)	
The healthcare providers adapted to my level of understanding, using appropriate language.	Clinic	26 (72.2)	1 (2.8)	9.0 (25.0)
ED	25 (69.4)	11 (30.6)	0 (0)	
The healthcare providers verbally expressed empathy.	Clinic	34 (94.4)	1 (2.8)	1 (2.8)
ED	30 (83.3)	6 (16.7)	0 (0)	
The healthcare providers encouraged me to ask questions.	Clinic	12 (33.3)	14 (38.9)	10 (27.8)
ED	27 (75.0)	5 (13.9)	4 (11.1)	
The healthcare providers discussed assessment and involved me in deciding upon a plan.	Clinic	16 (44.4)	18 (50.0)	2 (5.6)
ED	28 (77.8)	8 (22.2)	0 (0)	
The healthcare providers elicited my perspective and addressed any concerns I have about the plan. ^*^	Clinic	15 (41.7) ^**^	18 (50.0)	2 (5.6)
ED	26 (72.2) ^***^	8 (22.2)	0 (0)	

* N=36 for clinic and N=36 for ED

** 1 missing value

*** 2 missing values

### Facilitator feedback

All disciplines were involved in formulating and implementing the treatment plan with emphasis on their own professions’ specialty. However, the facilitator checklist recorded considerable overlap of students performing the same tasks ( Table 2).

**Table 2.** Summary of Facilitator Checklist Items for Combined Clinic and ED Teams

Checklist Item	Medical Student, N(%)	Pharmacy Student, N(%)	Nursing Student, N(%)	Not Completed
Obtain a focused history from the patient	36 (100)	7 (19)	31 (86)	0 (0)
Perform a focused physical examination	35 (97)	2 (6)	33 (92)	0 (0)
Develop the differential diagnosis	33 (92)	2 (6)	13 (36)	3 (8)
Order diagnostic tests	35 (97)	2 (6)	23 (64)	0 (0)
Diagnose ST elevation MI on EKG	35 (97)	3 (8)	17 (47)	0 (0)
Explain the diagnosis to the patient	36 (100)	4 (11)	22 (61)	0 (0)
Recognize need for higher level of care	36 (100)	12 (33)	31 (86)	0 (0)
Formulate an appropriate treatment plan	31 (86)	22 (61)	30 (83)	0 (0)
Discuss the treatment plan with the patient	35 (97)	9 (25)	32 (89)	1 (3)
Implement appropriate interventions	36 (100)	36 (100)	36 (100)	0 (0)
Reassure the patient about his condition	35 (97)	13 (36)	34 (94)	0 (0)

For instance, medical and nursing students over-lapped in history taking and physical examination. The medical students predominantly developed the differential diagnosis, ordered diagnostic tests and diagnosed ST-segment elevation myocardial infarction on electrocardiogram (EKG). The nursing students identified the need for a higher level of care, implemented the treatment plan and reassured the patient about their condition. The pharmacy students prepared the medications and implemented the treatment plan. As expected, nursing and medical students were more likely to explain the diagnosis to the patient and reassure the SP about their condition than the pharmacy students. Each multidisciplinary facilitator evaluated the student’s performance as a team, not as individuals ( Table 3).

**Table 3.** Summary of Facilitator Evaluation of Team Performance Using PACT Tool

PACT Component	Location N=53 ^*^	Poor	Below Average	Average	Above Average	Excellent	Not recorded
Team Structure	Clinic	3 (6)	3 (6)	17 (32)	11 (21)	15 (28)	4 (8)
ED	0 (0)	0 (0)	21 (40)	14 (26)	14 (26)	4 (8)
Leadership	Clinic	0 (0)	4 (8)	15 (28)	18 (34)	15 (28)	1 (2)
ED	1 (2)	2 (4)	14 (26)	23 (43)	12 (23)	1 (2)
Situational Monitoring	Clinic	0 (0)	5 (9)	9 (17)	17 (32)	21 (40)	1 (2)
ED	0 (0)	0 (0)	14 (26)	18 (34)	20 (38)	1 (2)
Mutual Support	Clinic	0 (0)	3 (6)	5 (9)	20 (38)	24 (45)	1 (2)
ED	0 (0)	1 (2)	9 (17)	17 (32)	24 (45)	2 (4)
Communication	Clinic	0 (0)	1 (2)	12 (23)	13 (25)	24 (45)	3 (6)
ED	0 (0)	1 (2)	8 (15)	19 (36)	12 (23)	13 (25)

* 53 facilitator evaluations included in this analysis, 1 missing evaluation.

On the first day of the event, facilitators completed one PACT tool for both clinic and ED teams. On the second day, the completed one for each team and results from the second day only are shown in Table 3 to compare the performance between clinic and ED teams (N=53 facilitators). Students performed better in mutual support and situational monitoring than on team structure or leadership. Facilitators rated student teams as excellent or above average in mutual support (83% clinic, 77% ED) and situational monitoring (72% clinic, 72% ED, Table 3). The facilitators rated 49% and 52% of student teams as excellent or above average for their team structure in the clinic and ED, respectively ( Table 3). For the communication domain, 70% and 59% of students were excellent or above average for the clinic and ED, respectively ( Table 3). The lower communication scores in the ED scenario likely reflect the lack of acute care experience, which detracted from the student’s ability to communicate with one another.

Student self-reflection using entrustable professional activities

Students evaluated their team performance using EPAs on a scale from 0-4 in four domains: Subjective, Objective, Assessment and Plan (See Appendix C) ( Table 4). Across all disciplines, students rated their teams on average as requiring light to no supervision with a median score of 2.5 or 3.0 on all four domains. However, the score varied from low to top score on most domains. Nursing students rated their team significantly higher than medical students across all domains and higher than pharmacy students in the objective and assessment domains. Scores from pharmacy and medical students were not statistically different across all domains.

**Table 4.** Student Self Reflection Using Entrustable Professional Activities

Domain	Nursing N=106Median (Range)	Pharmacy N=56Median (Range)	Medicine N=131Median (Range)	P Value
Subjective	3.0 (1.0-4.0)	3.0 (1.5-4.0)	2.5 (0.5-4.0)	<0.01
Objective	3.0 (1.0-4.0)	3.0 (1.0-3.5)	2.5 (0.5-4.0)	<0.01
Assessment	3.0 (1.0-4.0)	2.5 (1.0-4.0)	2.5 (0.0-4.0)	<0.01
Plan	3.0 (1.0-4.0)	3.0 (0.5-4.0)	2.5 (0.5-4.0)	0.02

Refer to Appendix C for full description of tool and rating scale.

## Discussion

This study designed a myocardial infarction clinical scenario of high risk and value for interprofessional cooperation between three health professions. Students from the three disciplines of medicine, nursing and pharmacy received feedback from the SP, facilitators representing all 3 professions, as well as peer and self-assessment. We believe that our development and implementation of a 360-Degree Performance Feedback System for IPE using SPs is a valuable method to increase reflective learning in the students. It also identifies gaps in knowledge and skills in our respective professional curricula. In the following, we discuss the learning outcomes from the perspective of the students, SP, and facilitators from different professions, as well as the strengths and weaknesses of the 360-Degree Performance Feedback System for IPE.

### Standardized patient feedback

Standardized patient feedback is valuable to assess the psychosocial and communication aspects of patient care provided by the students. This assessment allows the students to see how their actions, words and decisions affect the emotions and perceptions of a patient. Overall, scores given by the SPs were good. However, SPs noted that primary care student teams did not always explain information appropriate to their understanding or encourage the SPs to ask questions. Studies have shown that patients place high value on the interpersonal skills of providers as well as their medical knowledge. (Wagner *et al.*, 2007) Novice learners may struggle with prioritizing and multi-tasking in urgent medical conditions with limited time for patient interaction. Many of the learners have had considerable experience individually interacting with SPs. However, when placed in a team setting, the learners had to balance interacting with the SP, communicating with the team, and performing discipline-specific tasks.

This IPE scenario involved SPs who are professional actors and have experience working with medical students. SP feedback should reflect that of the patients they portray, but the use of professional SPs to evaluate novice students may create a disadvantage to students. As seen in Table 1, SPs somewhat felt that the students did not fully adapt to their level of understanding using appropriate language. However, these preclinical students are still learning the terminology of their professional language and were well aware that they are observed by the facilitators. Novice learners may not have the confidence required to receive and answer numerous questions from complex patients.

### Facilitator feedback

The facilitators critically appraised the clinical content delivered by the student teams. Most fundamental elements of the case were acknowledged and appropriately addressed by at least one member of the team. Student actions aligned within their discipline (i.e. medical and nursing students performed history-taking and physical exams, pharmacy students formulated and implemented the treatment plan). Students showed understanding of their discipline-specific roles but were not able to rectify overlapping responsibilities through task delegation. Facilitators noted that student teams were collaborative and collegial as evidenced by higher ratings in situational monitoring and mutual support. However, this was at the expense of team structure with poor delineation of roles and responsibilities as demonstrated by multiple medical and nursing students performing the same task on the patient. Although extra-curricular leadership opportunities exist within each discipline, students at this level rarely have opportunities to lead a healthcare team. These students struggled with identifying a group leader and those groups with a leader had difficulty carrying out the role.

### Student self-reflection

Student self-reflection allows students to make sense of what they have learned and how they can improve. Students felt they were ready for practice as evidenced by their high EPA scores. However, SPs noted a lack of effort to educate them on a serious medical condition. Additionally, facilitators noted a lack of team structure, leadership and suboptimal communication. Eva and colleagues have demonstrated this discrepancy between student evaluation and patient or facilitator evaluation, and suggest that novice learners must have a baseline level of confidence to be able to participate in this type of exercise ( Eva *et al.*, 2012). The nursing students felt more confident about their team performance compared to their peers. The nursing curriculum incorporates experiential training early in the first year of nursing school and second year nursing students are close to graduation. The nursing students have worked in the acute care setting and have experience being part of the healthcare team. However, they had no experience in the primary care setting. In contrast, the EPA scores were lower from medical and pharmacy students. These students demonstrated less confidence due to limited acute care experience prior to the IPE event. For students to further refine their team-based skills, multiple IPE events in different practice settings should be woven throughout each curriculum.

### Lessons learned from rubbing shoulders with peers

#### -The professional perspective

Without doubt, it is not only students who find it challenging to collaborate across professional boarders. We encountered numerous practical obstacles: finding time in already impacted teaching schedules, room facilities and commitment from 108 facilitators for two consecutive days. Each year we receive positive feedback from facilitators that participation in the IPE will likely positively impact their practice. They plan to seek ways to incorporate other disciplines in patient care. This “ripple effect” raises the possibility that IPE may improve patient outcomes in care provided by our facilitators. This simulation of a myocardial infarction in primary and secondary care provides opportunities for students to practice critical skills such as multitasking, aseptic medication preparation, using common terminology, and handling negative feedback from patients or peers. Through early engagement in patient care using simulation, students appreciate the importance and relevance of course materials before they are required to deliver care to real patients.

As a facilitator evaluating a team of interprofessional students, it can be frustrating and even embarrassing to watch one’s own students underperforming in knowledge and skills in front of others. This can lead facilitators to deflate student performance during debriefing sessions. We defined the scope and capabilities of the student learners to emphasize to facilitators that their role was to assess communication and team dynamics with less emphasis on scientific knowledge and skills. Students displayed a lot of positive energy and confidence as demonstrated by their team evaluations. Facilitators may view this as overconfidence, however, our role as educators is to remind facilitators that students require a significant amount of confidence to participate in performing new or unfamiliar skills within a complex, time-limited case.

#### -The student perspective

It takes time to build group dynamics, often months or years. Our students did not know each other prior to the event, so how realistic is the expectation that teams would function well minutes after being introduced to one another? Some groups were lucky to have a student leader emerge within minutes. Other groups struggled to delegate tasks, and therefore overlapped in areas such as history taking or physical assessment. In real life, teams rarely consist of all new members on any given day. It was therefore not surprising that the whole range of scores was used from poor to excellent when the facilitators assessed team structure, leadership, situational motoring, mutual support and communication ( Table 3).

Based on facilitator observations, student self-evaluation of their team performance with the highest possible scores was unanticipated. Was this a reflection of their lack of experience or their track record of success? In this scenario, nursing students who had more clinical exposure appeared more confident than the less experienced medical or pharmacy students. The medical students struggled with multi-tasking and situational monitoring and the pharmacy students with preparation of intravenous medications under time pressure. This further supports the need for offering students multiple IPE events in different practice settings to develop their team-based skills and confidence.

### Strengths and limitations

In other learning environments, the short and long term effect of the 360 model has been reported to be mixed, primarily due to the variability of assessment models. ( Bracken DW, 2011) A strength of this study was that our 360-Degree Performance Feedback System was based on modified version of published tools(Chiu, Brock and Abu-Rish; Wallace, 2007; May, 2008) and that we managed to tailor it for our high number of facilitators, SPs and students (Appendices A, B, C). A limitation of this study is that the students are novice learners with limited clinical experience. The SP were asked to score them on communication skills and not on clinical skills ( Table 1). The facilitators scored them as a *team* on clinical skills and the PACT Components ( Table 2 and 3), but not on *individual* performance. It is therefore possible that gaps in clinical knowledge on an individual level were not revealed. Also, our 108 facilitators had variable experience with simulation as a teaching method, and limited experience with interprofessional co-debriefing, the checklists and rating scores. Finally, inter-rater reliability testing was not performed on any of the tools.

## Conclusion

We believe that simulation technology highlights the importance and relevance of the curricula in a way that prepares students for challenges of delivering care to real patients. In this study, the implementation of a tailor made 360-degree feedback model with facilitator, SP, peer and student self-appraisal was useful to give comprehensive feedback to novice medical, nursing and pharmacy students. It also highlights knowledge gaps in the curricula within and between the respective health care professions. The 360-Degree Feedback Model has a generic format suitable for other institutions educating medical, nursing and pharmacy students.

## Take Home Messages

• Most pre-clinical health science students have little experience working on health care teams and require coaching on team structure, organization and dynamics.
• Including multiple perspectives from the expert teacher, patients and peers, enriches performance feedback for students.
• Educators can expect positive student self-appraisals or peer feedback given limited experiences of pre-clinical students.
• Patient satisfaction feedback is extremely impactful to pre-clinical students and imparts an emotional connection to the learning experience.

## Notes On Contributors

Linda Awdishu is an Associate Clinical Professor of Pharmacy and the Director of Simulation and Interprofessional Education at the University of California, San Diego Skaggs School of Pharmacy and Pharmaceutical Sciences. She is also an Associate Clinical Professor of Medicine at the University of California, San Diego School of Medicine.

Amy Zheng is a Senior Physician for the Veteran Affairs Office of Inspector General and Assistant Clinical Professor of Medicine, University of California, San Diego School of Medicine.

Anne Gerd Granas is a Professor of Pharmacy at the University of Oslo School of Pharmacy. At the time of manuscript prepration, she was a visiting Fulbright Scholar at the Univeristy of California, San Diego.

Janine Gallasso is a Clinical Pharmacist in the Department of Pharmacy, University of California, San Diego Health System.

Karen Macauley is the Associate Dean for Advanced Practice Programs and Professor of Nursing at the University of San Diego, Hahn School of Nursing and Health Science.

Cheryl Butera is an Associate Professor of Nursing and Director of Innovative Learning and the Dickinson Nursing Simulation Center at the University of San Diego, Hahn School of Nursing and Health Science.

Sophie Hutchins is an Associate Professor of Nursing at the Hahn School of Nursing and Health Science, University of San Diego.

Peggy Wallace is an Associate Professor of Medicine and Director of Simulation Education at the University of California, San Diego School of Medicne.

Karen Garman is the Regional Director of Graduate Medical Education at Kaiser Permanente Southern California.

Jennifer Namba is an Associate Clinical Professor of Pharmacy and Assistant Director of Simulation and Interprofessional Education at the University of California, San Diego Skaggs School of Pharmacy and Pharmaceutical Sciences

## References

[ref1] Bracken DW R. D. (2011) When Does 360-Degree Feedback Create Behavior Change? And How Would We Know It When It Does? J Bus Psychol. 26, pp.183–192. 10.1007/s10869-011-9218-5

[ref2] CAIPE (2002) Defining IPE. Available at: https://www.caipe.org/( Accessed: February 12, 2018).

[ref3] Chiu C.-J. Brock D. and Abu-Rish E . Center for Human Sciences Interprofessional Education, Research and Practice. Performance Assessment of Communication Tool Set. University of Washington. Available at: http://collaborate.uw.edu/educators-toolkit/tools-for-evaluation/performance-assessment-of-communication-and-teamwork-pact-too( Accessed: February 27, 2018).

[ref4] Ericsson K. A. (2004) Deliberate practice and the acquisition and maintenance of expert performance in medicine and related domains. Acad Med. 79(10 Suppl), pp.S70–81. 10.1097/00001888-200410001-00022 15383395

[ref5] Ericsson K. A. (2008) Deliberate practice and acquisition of expert performance: a general overview. Acad Emerg Med. 15(11), pp.988–94. 10.1111/j.1553-2712.2008.00227.x 18778378

[ref6] Eva K. W. Armson H. Holmboe E. Lockyer J. (2012) Factors influencing responsiveness to feedback: on the interplay between fear, confidence, and reasoning processes. Adv Health Sci Educ Theory Pract. 17(1), pp.15–26. 10.1007/s10459-011-9290-7 21468778 PMC3274671

[ref7] Garman A. N. Tyler J. L. and Darnall J. S. (2004) Development and validation of a 360-degree-feedback instrument for healthcare administrators. J Healthc Manag. 49(5), pp.307–21; discussion 321-2, 10.1097/00115514-200409000-00007 15499805

[ref8] Joshi R. Ling F. W. and Jaeger J. (2004) Assessment of a 360-degree instrument to evaluate residents’ competency in interpersonal and communication skills. Acad Med. 79(5), pp.458–63. 10.1097/00001888-200405000-00017 15107286

[ref9] May W. (2008) Training Standardized Patients for a High-Stakes Clinical Performance Examination in the California Consortium for the Assessment of Clinical Competence. The Kaohsiung Journal of Medical Sciences. 24(12), pp.640–645. 10.1016/S1607-551X(09)70029-4 19251559 PMC11917839

[ref10] O’Gara P. T. Kushner F. G. Ascheim D. D. Casey D. E. (2013) 2013 ACCF/AHA Guideline for the Management of ST-Elevation Myocardial Infarction A Report of the American College of Cardiology Foundation/American Heart Association Task Force on Practice Guidelines. Journal of the American College of Cardiology. 61(4), pp.e78–e140. 10.1016/j.jacc.2012.11.019 23256914

[ref11] Overeem K. Wollersheim H. C. Arah O. A. Cruijsberg J. K. (2012) Evaluation of physicians’ professional performance: an iterative development and validation study of multisource feedback instruments. BMC Health Serv Res. 12, p.80. 10.1186/1472-6963-12-80 22448816 PMC3349515

[ref12] Sargeant J. Armson H. Chesluk B. Dornan T. (2010) The processes and dimensions of informed self-assessment: a conceptual model. Acad Med. 85(7), pp.1212–20. 10.1097/ACM.0b013e3181d85a4e 20375832

[ref13] Schmidt R. A. and Wulf G. (1997) Continuous concurrent feedback degrades skill learning: implications for training and simulation. Hum Factors. 39(4), pp.509–25. 10.1518/001872097778667979 9473972

