# Supplementary material for: 360-Degree Feedback Model to Enhance Interprofessional Learning
Source: MedEdPublish (2016). 2018 Jul 25;7:154. [Version 1] doi: 10.15694/mep.2018.0000154.1 (PMC10701808; doi:10.15694/mep.2018.0000154.1)
Supplement: Peer review history

# Reviewer report: Reviewer response for version 1

**DOI:** 10.21956/mep.19617.r29171

**Published:** 2019-02-27

**Authors:** Ken Masters

**Affiliations:** 1 Sultan Qaboos University

This review has been migrated. The reviewer awarded 4 stars out of 5 A really comprehensive and detailed paper dealing with an important topic. The added appendices are appreciated: without breaking the flow of the narrative, the authors have been able to supply valuable detail to their paper.The other reviewers have already given detailed comments, so there is little to be served by raising those points, although, as has been pointed out, for future evaluations of this type, greater care might be taken when dealing with the pharmacy students, ensuring there is appropriate relevance of the information to them.Overall, a very valuable exercise and useful paper.

Reviewer Expertise:

NA

No decision status is available

# Reviewer report: Reviewer response for version 1

**DOI:** 10.21956/mep.19617.r29168

**Published:** 2019-02-25

**Authors:** Trevor Gibbs

**Affiliations:** 1 AMEE

This review has been migrated. The reviewer awarded 4 stars out of 5 An interesting paper to read although I would argue against its title, given the extensive use of the 360 degree assessment model- I don't feel that it quite fits in here and is not giving an unbiased assessment of the students.However, saying that I enjoyed reading the paper and I felt that it carried a lot of information regarding peer and SP assessment. For this reason I feel that it is a paper worth reading for all those involved i IPE.

Reviewer Expertise:

NA

No decision status is available

# Reviewer report: Reviewer response for version 1

**DOI:** 10.21956/mep.19617.r29170

**Published:** 2019-02-13

**Authors:** Ivana Guarrasi

**Affiliations:** 1 University of California, San Diego

This review has been migrated. The reviewer awarded 5 stars out of 5 Training and assessing interprofessional communication competencies present challenges due to the multifaceted and emergent nature of team collaboration. This study seeks to offer insights on how to improve assessment instruments in interprofessional education (IPE). Researchers rightfully point towards the importance of analyzing the debriefing stage of student training as a springboard for articulating, concretely and precisely, assessment instruments targeting communication and coordination of teamwork between nursing, medical and pharmacy students. The main contribution of this paper is the application of the 360-degree performance feedback in standardized patient (SP) simulations to render intelligible feedback parameters in the context of a complex, large-scale interprofessional training. While the study does provide insights on how to further develop training of IPE simulation, it does not include tools to trace the dynamic of interprofessional collaboration among students, considering its value for each participating profession. Further research would also necessitate feedback model aimed at training faculty mentors, incorporating what has been learned from the adoption of the 360-degree student feedback model.From the perspective of researchers working at the intersection of communication and science and technology studies, this study provides a window into new approaches to assessing IPE simulation training. It is an important stepping stone for establishing a baseline for a feedback model crafted in a multi-pronged manner. This research also yields the promise of fruitful collaboration with scholars working at the intersection of social sciences and medicine that draw on qualitative methods and deep ethnographic description of specific training events.Researchers grapple with the question of how to create training and assessment tools that are responsive to the complex nature of interprofessional team collaboration. This study provides a solid scaffold for further research, which would continue the work done in this paper.

Reviewer Expertise:

NA

No decision status is available

# Reviewer report: Reviewer response for version 1

**DOI:** 10.21956/mep.19617.r29169

**Published:** 2018-09-17

**Authors:** Crystal Zhou

**Affiliations:** 1 UCSF

This review has been migrated. The reviewer awarded 5 stars out of 5 The authors have attempted to assess the 360-Degree model of feedback in the interprofessional setting within a School of Medicine, Nursing, and Pharmacy during a simulated patient case scenario. The standardized patients and facilitators provided feedback to the students. There was also a self-reflection exercise and peer-to-peer feedback (not reported in the manuscript).Although this paper addresses an interesting topic that poses a gap in knowledge, I have the following comments to offer: For the methods section,o It is great that the students are receiving feedback from the standardized patients, facilitators, and their peers, however, the type of feedback seems to vary (e.g. facilitators are asking reflective questions, standardized patients are providing feedback on communication skills, and the student self-appraisal requires students to rank their performance based on EPAs). The type of feedback received allows the students to improve in various areas, however, doesn’t allow the students to compare their self-appraisal with a facilitator or standardized patient’s assessment with regards to EPAs. In essence, the feedback is not targeted and may overwhelm the student.For the results section,o In Table 2, it seems like most of the checklist items don’t necessarily pertain to pharmacy students (e.g. physical exam, order diagnostic tests, diagnose ST elevated MI); it may be more beneficial to create a discipline specific checklist for what each professional student is expected to accomplish during the encounter versus some student pharmacists completing a physical exam and others not completing one. This may confound the feedback received from the facilitators and standardized patients.o The authors note in the results that “pharmacy students prepared the medications and implemented the treatment plan,” but there is no checklist item in Table 2 to assess whether or not the pharmacy students prepared the medications appropriately and safely.For the discussion section,o The authors mention that the students struggled when there were overlapping roles between the professions; was there an initial team huddle where the students were able to discuss their individual roles? It seems like a leader emerged within some teams prior to the actual scenario, but this was not made clear.o It would be interesting to see what the students’ main take-home points were from this IPE exercise.Overall, I believe that this paper contributes significantly to the existing literature on providing effective feedback to learners in an IPE setting.

Reviewer Expertise:

NA

No decision status is available
